# In support of the placental programming hypothesis: Placental endocrine insufficiency programs atypical behaviour in mothers and their offspring

**DOI:** 10.1113/EP089916

**Published:** 2022-01-17

**Authors:** Rosalind M. John

**Affiliations:** ^1^ Cardiff School of Biosciences Cardiff University Cardiff UK

**Keywords:** fetal growth restriction, maternal depression, neurodevelopment, placental endocrine insufficiency

## Abstract

**New Findings:**

- **What is the topic of this review?**
  More than half of all pregnancies in the UK are exposed to adversity linked to increased problems in pregnancy for mothers and adverse outcomes for their children, but we do not know the mechanism(s) underpinning these relationships.
- **What advances does it highlight?**
  Studies in mice prove that placental endocrine insufficiency driven by genetic manipulation of imprinted genes in the offspring can concurrently drive fetal growth restriction, alterations in maternal caregiving and aberrant behaviour in wild‐type offspring exposed to an adverse environment. This suggests that placental endocrine insufficiency might contribute to the co‐morbidity of low birth weight, maternal depression and neurodevelopmental disorders observed in human populations.

**Abstract:**

Prenatal adversity, which is estimated to impact more than half of all pregnancies in the UK, compromises fetal growth and increases the chances of stillbirth, prematurity and infant mortality. Beyond these immediate and highly visible problems, infants that survive carry the invisible burden of increased risk of some of the most common and pervasive diseases that impact human populations. In utero exposure to depression and anxiety is one adversity that has been linked to these poorer outcomes, suggesting that maternal mood disorders drive the outcomes. However, recent studies in animal models suggest that both the maternal mood disorders and the detrimental outcomes for children could be the result of the same underlying placental pathology. In these studies, genetically wild‐type rodent mothers exposed to placental endocrine insufficiency engaged in less pup‐focused behaviours and less self‐care. Genetically wild‐type rodent offspring raised in this abnormal environment exhibited increased anxiety‐like behaviours, with male offspring additionally exhibiting deficits in cognition and atypical social behaviour, with some evidence of depressive‐like symptoms. This work establishes experimentally that placental endocrine insufficiency alone is sufficient to drive atypical behaviour in both mothers and their offspring. Although there are some data to suggest that this phenomenon is relevant to human pregnancy, considerably more work is required.

## 1 INTRODUCTION

More than half of all pregnancies in the UK are impacted directly by prenatal adversity, with many exposed to multiple adversities. A recent survey in the UK reported that 27.4% of pregnant women were overweight, 18.3% obese and 3.3% severely obese at their first booking appointment, which takes place in the first trimester of pregnancy (Public Health England data, 2017). Excessive weight gain in pregnancy, an indicator of overnutrition, is estimated at 47–56.0% (Garay et al., 2021; Goldstein et al., 2017).

Despite the prevalence of overnutrition, pregnancy is also an especially vulnerable time for dietary deficiencies owing to the extraordinary nutritional demands of the fetus and placenta. Although 85.8% of women reported taking a folic acid supplement before or on confirmation of pregnancy (NHS Statistics England, 2019–2020), this still leaves 14.2% potentially deficient in this crucial nutrient known to protect against neural tube defects. Vitamin D deficiency, which affects black and minority ethnic groups disproportionately, is thought to impact up to nearly half of pregnant women (Palacios et al., 2019). Women with vitamin D deficiency are more likely to have pregnancy complications including pre‐eclampsia, diabetes, preterm birth and small babies, with some evidence that vitamin D supplementation protects against low birth weight (Maugeri et al., 2019). In addition to these dietary adversities, one in four women will develop some form of mental health issue during pregnancy and the months after childbirth, with escalating rates in younger women (Pearson et al., 2018).

**FIGURE 1:**
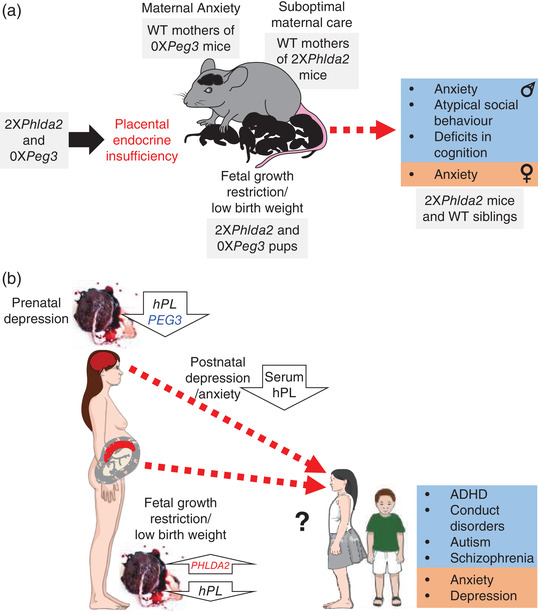
The placental programming hypothesis. (a) Summary of findings from mouse models in which wild‐type dams exposed to placental endocrine insufficiency exhibit altered maternal behaviour, and wild‐type offspring exposed to an adverse environment induced by placental endocrine insufficiency exhibit alterations in their behaviour. Note: 0X refers to loss of expression and 2X refers to two‐fold increased expression. (b) Summary of the indirect evidence from human studies that placental endocrine insufficiency driven by the aberrant expression of imprinted genes in the placenta might explain the co‐occurrence of maternal mood disorders and adverse outcomes for children. Abbreviations: hPL, human placental lactogen; *Peg3* or *PEG3*, paternally expressed 3; *Phlda2* or *PHLDA2*, pleckstrin homology like domain family A member 2; WT, genetically wild‐type. Note that this figure summarizes work presented at the Physiology Online 2021 symposium

Numerous studies have shown that adversities such as these during pregnancy can contribute to substantially poorer lifelong outcomes for offspring, a phenomenon described as fetal programming or developmental origins of disease (Barker, 1990; Gluckman & Hanson, 2006). Some of the same adversities that drive poor outcomes for offspring have also been linked to changes in maternal caregiving in animal models, another factor key to ensuring the lifelong health of offspring (R. M. John, 2019). Disentangling the mechanisms behind these relationships is challenging. However, recent studies using animal models suggest a novel mechanism, which is placental endocrine insufficiency (Figure 1), that links adversity in pregnancy to both abnormal maternal care and abnormal offspring behaviour.

The placenta is an extraordinary tissue, essential for prolonged development in utero (R. John & Hemberger, 2012). The placenta functions as a sophisticated transportation system, channelling nutrients to support the growing fetus and providing a physical barrier between the maternal and fetal circulation. The placenta is also a major endocrine organ, flooding the maternal circulation with hormones that induce adaptations in the mother required for a successful pregnancy. Although many of these adaptations ensure nutrient availability and prevent immune rejection, one less well‐recognized adaptation involves the priming or programming of maternal behaviour during pregnancy.

Most well studied in rodents, changes occur to the maternal brain to prepare the new mother for her role as a caregiver. Although virgin female rodents and, to some extent, males can acquire parental behaviour, even in some cases simply by copying the behaviour of new mothers (Carcea et al., 2021), it is only after experiencing a pregnancy that females respond immediately to pups.

Hormones key to the priming of maternal behaviour are members of the prolactin/growth hormone family (Smiley et al., 2019). Both prolactin expressed by the pituitary and placental lactogens expressed by the placenta can stimulate maternal behaviours when infused directly into the brains of non‐pregnant females (Smiley et al., 2019). Heterozygous ablation of the maternal prolactin receptor, which binds these hormones, results in deficits in maternal caregiving (Smiley et al., 2019). In both humans and rodents, placental lactogens are expressed from specialized endocrine lineages in direct contact with maternal blood (haemochorial). In humans, these are the syncitiotrophoblast and extravillous trophoblast (Liu et al., 2018), whereas in mice they are called the spongiotrophoblast and the trophoblast giant cells (Simmons et al., 2008). Genes that regulate the development of these placental endocrine lineages, in addition to environmental factors that influence hormone secretion, therefore have potential to influence maternal caregiving behaviour by modulating exposure of the maternal brain to placental lactogen.

## 2 IMPRINTED GENES AND THE ENDOCRINE FUNCTION OF THE MOUSE PLACENTA

Imprinted genes are expressed from one parental allele as a consequence of epigenetic events initiated in the germline and propagated during early development to generate imprinted domains containing both maternally and paternally expressed genes (Surani, 1998). Although some silent alleles are directly spanned by DNA methylation inherited from the germline, the majority are not. This might render them more vulnerable to external factors. This is an important point, because a number of imprinted genes have been demonstrated experimentally to influence fundamentally important processes including fetal growth, placental development, metabolism and behaviour (Tucci et al., 2019). We discovered that imprinted genes converge to regulate the development of the placental endocrine lineages in mice.

The mature mouse placenta consists of three compartments: (1) the decidua, which is the maternally derived compartment formed from remodelling of the uterine wall; (2) the fetally derived junctional zone, which is the endocrine compartment composed of spongiotrophoblast and glycogen cells; and (3) the fetally derived labyrinth, which is the transport region, composed of a two‐layer syncitiotrophoblast covering the fetal capillaries (Woods et al., 2018). Throughout these compartments and in close contact with maternal tissue and vasculature are the trophoblast giant cell lineages (parietal, spiral artery, channel, sinusoidal and canal; Woods et al., 2018).

Studies in mice demonstrated that the maternally expressed imprinted genes pleckstrin homology‐like domain family A member 2 (*Phlda2*) (Frank et al., 2002; Salas et al., 2004; Tunster et al., 2010; Tunster, Creeth, et al., 2016), achaete‐scute family bHLH transcription factor 2 (*Ascl2*, otherwise known as *Mash2*) (Guillemot et al., 1995; Oh‐McGinnis et al., 2011; Tanaka et al., 1997; Tunster, McNamara, et al., 2016) and cyclin‐dependent kinase inhibitor 1C (*Cdkn1c*) (Takahashi et al., 2000; Tunster et al., 2011) all encode repressors of the spongiotrophoblast lineage. *Ascl2* additionally limits the number of parietal trophoblast giant cells. *Cdkn1c* is required for sinusoidal trophoblast giant cell differentiation. In contrast, loss of expression of paternally expressed 3 (*Peg3*) resulted in a significant loss of both spongiotrophoblast and glycogen cells (Tunster et al., 2018). Essentially, ‘paternalization’ (silencing of the paternal allele) appears to secure higher maternal investment by increasing the production of placental hormones by the fetal components of the placenta, whereas ‘maternalization’ counteracts to protect maternal reproductive fitness, consistent with the idea that genomic imprinting evolved in response to differences in the allocation of parental resources by male and female mammals (Haig, 1993). As with maternal nutrients, maternal care might be a resource over which the parental genomes are in conflict.

## 3 *Phlda2* AND MATERNAL CAREGIVING

*Phlda2* is maternally expressed in the ectoplacental cone where the progenitors of spongiotrophoblast reside (Dunwoodie & Beddington, 2002; Frank et al., 1999). A twofold increase in expression of *Phlda2* results in a 50% loss of spongiotrophoblast and late, asymmetrical fetal growth restriction (Salas et al., 2004; Tunster et al., 2010, 2014; Tunster, Creeth, et al., 2016), whereas loss of expression results in a heavier placenta, with an approximate doubling of spongiotrophoblast (Frank et al., 2002; Tunster, Creeth, et al., 2016). By regulating the number of spongiotrophoblast cells that express placental hormones, *Phlda2* modulates the amount of hormones produced by the placenta. One key hormone expressed by the spongiotrophoblast is mouse placental lactogen, encoded by prolactin family 3, subfamily b, member 1 (*Prl3b1*; previously named *PL‐II*) (Simmons et al., 2008). Consequently, mouse mothers (dams) carrying offspring expressing different doses of *Phlda2* are exposed to different levels of placental hormones.

If, as has been suggested previously, placental lactogens instruct maternal behaviour, this raises the possibility that the offspring might influence their mother's caregiving behaviour through the expression of imprinted genes in their placenta. To test this hypothesis, we set up three cohorts of dams carrying offspring with either loss of expression, normal expression or twofold overexpression of *Phlda2*, with the dams exposed to high, normal or low levels of placental hormones, respectively. Key to this experiment, pregnant dams were generated by the transfer of pre‐implantation embryos into pseudopregnant wild‐type females. This step ensured that the dams were genetically identical, meaning that any changes in their brains or behaviour could be attributed to the genotype of the offspring they carried. We identified alterations in maternal hypothalamic and hippocampal transcriptomes impacting pathways associated with G protein‐coupled receptors, olfactory transduction and gonadotrophin‐releasing hormone signalling 4 days before the dams gave birth (Creeth et al., 2018).

In a second cohort of dams, we assessed maternal behaviour using classical tests and observed the dam's behaviour toward her pups. Dams exposed to high placental hormones were slow to retrieve their pups and performed less well in a nest‐building task but spent significantly more time nursing and grooming their pups and engaging in self‐care. In contrast to dams exposed to levels of placental hormones higher than normal, dams exposed to levels lower than normal (placental endocrine insufficiency) were highly effective in the nest‐building task, performing even better that the control dams exposed to normal levels of placental hormones. However, these dams neglected both their pups and themselves during this task. Effectively, in the disturbed situation (nest‐building task) the dams exposed to higher levels of placental hormones in pregnancy prioritized caring for their pups and self‐care, whereas those exposed to lower levels of hormones prioritized rebuilding their nests. As an important point, dams exposed to higher levels of placental hormones continued to prioritize caring for their pups even when their pups were replaced by fully wild‐type foster pups, proving experimentally that their enhanced nurturing behaviour was programmed during pregnancy.

## 4 *Peg3* AND MATERNAL ANXIETY

*Peg3* is a paternally expressed imprinted gene originally made famous for its role in directly influencing maternal behaviour, with mutant dams neglecting their pups (Li et al., 1999). *Peg3* is also able to influence maternal behaviour indirectly when the mothers are wild‐type and the pups are mutant (McNamara et al., 2018). Loss of expression of *Peg3* in the placenta results in a 50% loss of spongiotrophoblast, at least in male placenta (Tunster et al., 2018), similar to the loss seen in response to a twofold increase in expression of *Phlda2* (Tunster et al., 2010; Tunster, Creeth, et al., 2016). Loss of *Peg3* additionally results in a 40% loss of glycogen cells, which also express some placental hormones, although not *Prl3b1* (Simmons et al., 2008). Wild‐type dams carrying and caring for *Peg3* mutant pups show altered behaviour, albeit somewhat different to that observed in response to a twofold increase in expression of *Phlda2*. Dams pregnant with mutant *Peg3* offspring showed subtle changes in their behaviour when first moved into a new cage mid‐way through pregnancy, but the major changes in behaviour were observed after birth, with these dams being slower to sniff and retrieve their pups and exhibiting anxiety‐like behaviour. A confounder to the interpretation of these observations is that *Peg3* mutant pups vocalize less, and pup vocalization is known to influence pup retrieval. It is therefore not possible to attribute either the delayed pup retrieval or the maternal anxiety to the prenatal influence of the placenta in this model.

## 5 PLACENTAL ENDOCRINE INSUFFICIENCY AND LATER LIFE BEHAVIOUR

In our *Phlda2* model where increased expression of *Phlda2* drives placental endocrine insufficiency, pups are born with low birth weight (Tunster et al., 2014). They are also neglected, at least when the dam is distracted (Creeth et al., 2018). Nonetheless, they still catch up to be comparable in weight to their wild‐type littermates, undergoing rapid catch‐up growth (Tunster et al., 2014). Being born small and being exposed to suboptimal maternal care are both associated with poorer behavioural outcomes for offspring, raising the possibility that these pups might be programmed to develop behavioural problems later in life.

In order to distinguish between the direct effect of genetically elevated *Phlda2* and the indirect effect of an adverse environment, we generated litters in which half the pups carried the *Phlda2* transgene (low birth weight plus adverse environment; transgenic) and half were genetically wild‐type but raised in the same adverse environment (which we refer to as non‐transgenic). These offspring were studied concurrently alongside fully wild‐type litters raised by wild‐type dams as environmental controls (Harrison et al., 2021). Both male and female offspring were put through a battery of behavioural tests to probe anxiety‐associated behaviours, depression‐like behaviours, aspects of learning and attention, and social behaviours. Through these tests, subtle but significant differences in the behaviours of offspring were uncovered. Specifically, transgenic and non‐transgenic offspring of both sexes showed evidence of heightened anxiety. In all other tests, experimental females did not differ from control females. However, transgenic and non‐transgenic males had detectable cognitive deficits, and transgenic males additionally exhibited atypical social behaviour, with mild indications of depressive‐like behaviours.

In addition to quantifying the behavioural consequences of placental endocrine insufficiency in this model, we also undertook a molecular approach by applying RNA sequencing analysis to four key brain regions, namely the adult hypothalamus, hippocampus, amygdala and ventral striatum. This analysis revealed striking differences in the transcriptional signatures between the groups. Of particular interest, we identified changes in gene expression unique to the transgenic animals in the hypothalamus and amygdala, which are brain regions that develop predominantly prenatally, when there are significant deficits in the placental spongiotrophoblast compartment (Tunster et al., 2010; Tunster, Creeth, et al., 2016) and when fetal growth is restricted (Tunster et al., 2014) in our model. In contrast, the hippocampus and ventral striatum, which continue to develop postnatally, were impacted in both the transgenic and non‐transgenic animals, suggesting that these regions might be responding to adversities experienced in the postnatal period. It is therefore tempting to speculate that the hippocampus and ventral striatum were impacted by the quality of maternal care. However, to test this formally would require cross‐fostering of animals, which was not done in this study. Nonetheless, this study provided the first evidence that placental endocrine insufficiency driven by the altered expression of an imprinted gene can cause alterations in the behaviour of genetically wild‐type offspring.

Other studies have identified specific hormones dependent on the placenta for their synthesis that play key roles in fetal brain development (Bonnin et al., 2011; Vacher et al., 2021), highlighting the crucial importance of individual placental hormones for offspring behaviour. Together, these studies firmly establish an active role for the placenta in shaping brain development, beyond that involved in supporting fetal growth.

## 6 PRENATAL ADVERSITY AND PLACENTAL ENDOCRINE DYSFUNCTION

Our studies in mice demonstrate formally that defective placental development impacting the production of placental hormones can concurrently drive low birth weight and alterations in the behaviour of both mothers and their offspring. There are many scenarios that can lead to defects in the placental endocrine lineages in mice. We have shown that, individually, genetic alteration to the imprinted genes *Phlda2*, *Cdkn1c*, *Ascl2* and *Peg3* alters the composition of the mouse placental endocrine compartment. Several other imprinted genes might function in a similar manner as positive and negative regulators of placental endocrine function (R. M. John, 2013), in addition to non‐imprinted genes.

Imprinted genes are of particular interest in this respect because they are known to respond epigenetically to prenatal adversities. For example, loss of silencing and hypomethylation of the paternal allele of *Cdkn1c* occur in utero when dams are fed low‐protein diets during pregnancy (Van de Pette et al., 2017). There are also a number of prenatal adversities linked to changes in the expression of imprinted genes and placental hormones, as reviewed by R. John (2019) and reported most recently for *Phlda2* (Eaton et al., 2020).

These experiments in animal models provide support to the idea that adversity in pregnancy programs changes to the offspring by inducing placental endocrine insufficiency. This idea does not exclude alterations to the fetal epigenome, as demonstrated formally to occur in response to a maternal low‐protein diet (Van de Pette et al., 2017), but could explain the co‐occurrence of suboptimal maternal caregiving and alterations in offspring behaviour observed in many models of prenatal adversity (R. John, 2019).

## 7 RELEVANCE TO HUMAN PREGNANCY

Numerous epidemiological studies have reported on the co‐morbidity of maternal mood disorders, low birth weight and behavioural disorders in children including depression, anxiety, attention deficit hyperactivity disorder and conduct disorders (Lautarescu et al., 2020).  The assumption has been that maternal mood disorders drive poor health outcomes in the children, and this will be the case for some pregnancies. However, a causal relationship between prenatal adversity and placental endocrine dysfunction has been established in several studies in animal models (Eaton et al., 2020; R. John, 2019).

We have demonstrated that placental endocrine insufficiency can cause low birth weight (Salas et al., 2004; Tunster et al., 2010, 2014; Tunster, McNamara, et al., 2016) and alterations in the behaviour of the mother (Creeth et al., 2018; McNamara et al., 2018) and her offspring (Harrison et al., 2021) in mice (Figure 1a). These studies in experimental animal models identify an alternative mechanism whereby adversity‐driven placental endocrine insufficiency causes both the mood disorder in the mother and the poor health outcome for the child.

There is some indirect evidence to suggest that this alternative mechanism is relevant to human pregnancy. For example, placental expression of *PEG3* is lower in pregnancies where mothers are clinically depressed or self‐report report higher symptoms of depression (Janssen et al., 2016), and several studies have reported associations between members of the prolactin/growth hormone family and maternal depression (Creeth & John, 2020) (Figure 1b). Importantly, low placental lactogen in maternal serum at term has been associated with higher symptoms of both depression and anxiety in mothers (Sumption et al., 2020). Low levels of placental hormones might result from adversity‐driven aberrant expression of imprinted genes in the placenta, causing defects in the development of the placental endocrine lineages. Alternatively, there might be a direct impact on gene expression, as has been suggested by studies on maternal obesity (Cattini et al., 2020). In the study by Cattini et al. (2020), reduced placental lactogen was associated with altered chromosomal confirmation at the locus spanning the gene, suggesting changes at the level of gene expression rather than the composition of the placenta.

It is likely that there are several routes by which adversity in pregnancy might drive placental endocrine insufficiency. The important point is that the outcomes will be common owing to the fundamental importance of placental lactogen for both maternal and fetal health. Previous research has focused primarily on the passive role of the placenta as a barrier protecting the fetal brain from damaging exposures. Our studies, and those of other groups, identify a more active role for the placenta in determining maternal and fetal brain health. Focused studies are now required to establish whether the influences of placental hormones on maternal and offspring behaviour demonstrated in experimental models have validity in human populations.

## COMPETING INTERESTS

None declared.
